# Studies in medicine during the COVID-19 pandemic in Kenya and germany: a cross-sectional pilot study

**DOI:** 10.1186/s12909-025-08198-0

**Published:** 2025-10-31

**Authors:** Allan Omollo, Cecilie Posingis, Pamela Godia, Andrea Budnick

**Affiliations:** 1 Department of Public and Global Health, University of Nairobi, Nairobi, Kenya; 2 Institute of Medical Sociology and Rehabilitation Science, Charité – Universitätsmedizin Berlin, corporate member of Freie Universität Berlin and Humboldt Universität zu Berlin, Berlin, Germany

**Keywords:** Students, Medicine, Kenya, Germany, COVID-19, Online learning, Depression, Anxiety

## Abstract

**Background:**

The COVID-19 pandemic disrupted global medical training and resulted in substantial alterations in the psychosomatic health of medical students. We conducted a comparative evaluation across diverse socio-cultural and economic settings in Kenya (LMIC) and Germany (HIC) to examine the changes wrought to students during the COVID-19 pandemic, their experiences and responses to ease the pandemic burden. We also assessed their attitudes towards the online training model that was introduced during the COVID-19 pandemic with an aim to develop effective educational approaches that could be adopted in learning institutions and offer support in future unpredictable events.

**Methods:**

We conducted a retrospective cross-sectional web survey three years post the announcement of the first COVID case among undergraduate medical students at the University of Nairobi, Kenya (UoN) and at Charité – Universitätsmedizin Berlin, Germany. Questionnaires were used to assess COVID-19-specific changes in the learning situation, evaluation of mental burden, satisfaction with the online learning (OL), and coping mechanisms of the students. Binomial and multiple regression analyses were applied.

**Results:**

In total, 122 undergraduate medical students participated in our cross-sectional pilot study (response rate: 78.71%). Respondents were from the UoN (65.5%) and Charité (34.4%). Kenyan and German students experienced similar levels of depression (PHQ-2^COVID^) (M = 2.12; SD = 1.36 and M = 2.02; SD = 1.22, *P =* 0.960) and anxiety (GAD-2^COVID^) (M = 2.09; SD = 1.54 and M = 1.91; SD = 1.59, *P* = 0.540) during the COVID-19 pandemic. For Kenyan students (M = 3.12; SD = 0.60) the transition to OL during the COVID-19 pandemic seemed to be a more comfortable way of learning, compared to the German students (M = 2.91; SD = 0.61). However, the OL score difference was not statistically significant (*P* = 0.078). In regards to mental health during the pandemic, most students from both countries attributed social support from either family, friends, or religious institutions as a benefactor in coping (UoN: 50.0%, Charité: 59.5%). Most of the students from UoN (40.0%) identified with hobbies such as movies, reading, writing, and social media, while German students (33.3%) identified with sporting activities such as gym, walks and workouts.

**Conclusion:**

There was significant negative impact on both medical training and the psychosomatic state of medical students in the survey. Such a comparative approach provides insights that isolated single-country studies cannot, thereby contributing to global medical education policy and guiding context-appropriate interventions for future crises.

## Introduction

On March 11, 2020, the World Health Organization (WHO) formally designated the unique COVID-19 virus to be a global pandemic. The majority of sectors, including the education and healthcare sectors, were significantly impacted by this. Medical students’ education was disrupted and their social, physical, and mental wellbeing were adversely affected by the partial or total lockdown of some countries, social isolation, and other measures implemented in an effort to stop the virus’s spread [1]. According to Struthers et al. the measures put in place to curb the spread of the virus globally involved suspending in-person lectures, limiting ward interactions with patients, and a rapid shift to online instruction. The sudden transition to virtual platforms interrupted the ongoing academic calendars, research projects and progression from one academic year to another [2]. Exams, regularly scheduled classes were all inevitably cancelled, and resulted in the shift from in-person instruction to online instruction [3]. The transition from one year to the next and the entry into post-graduate training programs were both delayed by this interruption. A number of active research sessions and projects were also suspended [2]. The effectiveness and efficiency of the online learning platforms as they relate to medical education were not thoroughly investigated, which had a detrimental impact on the training’s quality [4]. Students in various locations struggled with adapting to online instruction in addition to dealing with electrical shortages and bad network problems, which made it even harder for them to keep up with the virtual schoolwork and assignments. As well as increasing instances of burnout and weariness related to online learning platforms, students from disadvantaged backgrounds were unable to use the platforms due to the associated costs [4]. Several students were infected with the feared illness, were hospitalized, and required clinical management, hence missing out on learning in the process [5]. The medical training process became a less important agenda item in most healthcare and learning institutions as a result of the rising demand for health care services and the diversion of most resources to stop the virus’ spread [4]. Many studies have been conducted in an effort to document and improve readiness in the event of future occurrences mirroring this global pandemic because of the worry, loneliness, and disorientation that medical students faced during the COVID-19 pandemic [3]. It is significant to remember that the pandemic’s diminished opportunities for medical students to interact with patients, physicians, and their peers affected the trainees’ general wellbeing in both short- and long-term ways [1]. Students’ psychosomatic states can change during a pandemic due to social withdrawal, self-isolation, financial stress, scholastic difficulties, worries about the health of family members, and excessive social media use [5]. The COVID-19 pandemic undoubtedly affected medical students’ education and mental health during the shift to online learning according to Struthers et al. Nevertheless, online learning has been chosen as the best compromise to continue offering education during the pandemic era. The academic lives of students had been greatly impacted by multiple variables, such as changes in educational content and modifications to the structure of how students learn and college support, alterations in student-to-teacher, student-to-school, and student-to-student associations, the chance to develop a variety of skills, and financial issues brought on by the pandemic [5]. The COVID-19 pandemic caused significant suffering and mental burden for medical students in Kenya and Germany, highlighting the necessity for continued counseling and educational support for these students both during and after the pandemic [6, 7]. Wafula and Ong’era [7] published an experience report about the situation for medical students in Kenya but sufficient empirical research is lacking. Kenya is a collectivist society and a lower-middle-income country (LMIC) where technological resources, mental health services, and internet access are comparatively limited [8]. Germany is an individualist society and a high-income country (HIC) with robust institutional support and advanced digital infrastructure [9]. This deliberate choice of two contrasting contexts allows for a deeper understanding of how cultural and structural factors shape medical students’ experiences of pandemic-driven educational disruption. By examining both differences and similarities, we can distinguish challenges that are universal from those that are context-specific. This is critical for formulating strategies that are both adaptable to diverse environments and sensitive to local realities. Such a comparative approach provides insights that isolated single-country studies cannot, thereby contributing to global medical education policy and guiding context-appropriate interventions for future crises. Thus, the goal of this study is to understand undergraduate medical students’ experiences and challenges in Nairobi, Kenya and Berlin, Germany during the COVID-19 pandemic in order to better manage online learning and provide support in the event of future unpredictable situations. The research attempts to answer the following questions:

1. How do undergraduate medical students in Nairobi, Kenya and Berlin, Germany describe their psychosomatic status and their attitudes towards online learning during the COVID-19 pandemic?
2. What were the experiences among undergraduate medical students in Nairobi, Kenya and Berlin, Germany in their academic life in terms of beneficial factors for mental health and coping techniques (stress relieving activities) during the COVID-19 pandemic?
3. Are medical students’ attitudes towards online learning associated with their psychosomatic status and is there a difference between students from both countries?

## Methods

### Study population

We conducted a pilot retrospective web-survey among fourth to sixth year medical students at University of Nairobi in Kenya and at Charité – Universitätsmedizin Berlin in Germany between 06/23/2023 and 08/22/2023. The web-survey is part of the “Berlin – Nairobi Global HEART – Health Research, Exchange and Training” in the funding program “PAGEL - Partnerships for the health sector in developing countries 2021–2024” of the German Academic Exchange Council from 2021 to 2024 (Grant ID 57562598). The sample contains 122 students (*n* = 80 from University of Nairobi and *n* = 42 from Charité). A SoSci survey questionnaire was sent to medical students on school emails. We received responses from 155 undergraduate medical students. For level of study and the numbers of students at each institution see Table 1. Responses from 33 respondents were excluded due to missing data or they met the exclusion criteria which included non-medical students, under 18 years old and students not in level four to level six. The final sample, a convenience sample, thus had 122 students for analyses (response rate: 78.71%).

**Table 1:** Sociodemographic characteristics for medical students from university of Nairobi *(UoN)*, Kenya and Charité – Universitätsmedizin Berlin, Germany in 2023

Variable	Characteristics	Total (*N*, %)	UoN (*n*, %)	Charité (*n*, %)	*P*
Participants		122 (100.0)	80 (65.6)	42 (34.4)	
Sex	Male	47 (38.5)	36 (45.0)	11 (26.2)	0.062
	Female	71 (58.2)	43 (53.8)	28 (66.7)	
	Prefer not to say	2 (1.6)	1 (1.3)	1 (2.4)	
	Divers	2 (1.6)	0 (0.0)	2 (4.2)	
Year of Study	4	43 (35.2)	20 (25.0)	23 (54.8)	0.004
	5	49 (40.2)	38 (47.5)	11 (26.2)	
	6	30 (24.6)	22 (27.5)	8 (19.0)	
Living Conditions	Alone	15 (12.3)	4 (5.0)	11 (26.2)	0.000
	With Family	87 (71.3)	75 (93.8)	12 (28.6)	
	With Friends	15 (12.3)	1 (1.3)	14 (33.3)	
	Other, please specify	5 (4.1)	0 (0.0)	5 (11.9)	
Part time job	Healthcare Organization (COVID-19)	17 (13.9)	1 (1.3)	16 (38.1)	0.000
	Healthcare Organization (non-COVID-19)	18 (14.8)	5 (6.3)	13 (31.0)	
	Non-medical	15 (12.3)	11 (13.8)	4 (9.5)	
	No part-time job	72 (59.0)	63 (78.8)	9 (21.4)	
Volunteering	Yes	33 (27)	16 (20.0)	17 (40.5)	0.019
	No	89 (73)	64 (80.0)	25 (59.5)	
COVID-19 Diagnosis	PCR and IgM/IgG positive or clinically confirmed	38 (31.1)	8 (10.0)	30 (71.4)	0.000
	Suspected (Contact/asymptomatic)	27 (22.1)	23 (28.8)	4 (9.5)	
	No diagnosis	57 (46.7)	49 (61.3)	8 (19.0)	

Before commencement of data collection, ethical approval was obtained from Ethical review committee of the Kenyatta National Hospital - University of Nairobi Ethics and Research Committee, Kenya (Ref: KNH-ERC/UA/229). Administrative permit was provided from Charité – Universitätsmedizin Berlin, Germany to collect data from the medical students as this study was part of their curriculum requirements. Written consent regarding study information, anonymous survey, and data protection regulations was obtained from each study participant.

## Measurements

### Socio-demographic characteristics

The socio-demographic data included gender (male/female/genderfluid, current year of study (4/5/6), learning institution (University of Nairobi/Charité-Universitätsmedizin Berlin). We queried further characteristics: personal living situation at the time of the COVID-19 pandemic (alone/with parents/with own family/with other relatives/with friends), part-time job (Healthcare Organization – COVID-19/Healthcare Organization non-COVID-19/non-medical job/no part-time job), and COVID-19 diagnosis during the COVID-19 pandemic (clinically confirmed/suspected/no diagnosis) [5].

### Depression and anxiety

Depression and anxiety were measured with the German version of the Patient Health Questionnaire-4 (PHQ-4). The screening instrument which is ultra-brief consists of a two items depression scale (PHQ-2) and a two-items anxiety scale [Generalized Anxiety Disorder-2 (GAD-2)] and originally measures the amount of depression and anxiety symptoms an individual has felt during the past 2 weeks [10, 11]. The PHQ-2 contains the first two items of the PHQ-9 by using a 4-point Likert scale ranging from zero (not at all) to three (nearly every day). The PHQ-2 score ranges from 0 to 6. A score of 3 was identified as the optimal cut-off value when using the PHQ-2 to screen for depression. If the score is ≥ 3, major depressive disorder is likely. Patients who screen positive should further be evaluated for a depressive disorder. In addition, we implemented the General Anxiety Disorder assessment (GAD-2) to measure the level of anxiety [6]. The GAD-2 is also a screening tool using the 4-point Likert scale ranging from zero (not at all) to three (nearly every day). The score ranges from 0 to 6. A cut-off value of ≥ 3 was suggested to detect clinically relevant levels of anxiety symptoms. The GAD-2 is widely used to screen for general signs of anxiety. PHQ-2 and GAD-2 in combination add up to the PHQ-4, an ultra-brief screening instrument of depression and anxiety symptoms. The total score measures the symptom burden using the following categories: 0–2 (normal), 3–5 (mild), 6–8 (moderate) and 9–12 (severe) [6]. The PHQ-4 has been modified to investigate the period of time during the COVID-19 pandemic rather than only the previous two weeks. The modified temporal reference was integrated into the questions as follows; for the PHQ-2 the items read: *“During the COVID-19 pandemic did you feel little interest or pleasure in doing things?”* and *“Did you feel low*,* depressed*,* or hopeless during the COVID-19 pandemic?*”. For the GAD-2 the items read: *“Did you feel nervous/anxious/on edge with the changes brought about by the pandemic?”* and *“Were you able to stop or control worrying associated with the pandemic?”.* Due to these alterations, we renamed the scales into PHQ-2^COVID^ and GAD-2^COVID^ and tested their reliability and concurrent validity. In the present sample, for the medical students from UoN and from Charité the Cronbach’s alpha of the PHQ-2^COVID^ was 0.701 and 0.787 and for the GAD-2^COVID^ it was 0.780 and 0.808. The scales used, originate from established validated scales and were adapted to the COVID-19 situation by the interdisciplinary author team consisting of medical students, a social scientist, and a public health scientist. The PHQ-2^COVID^ and GAD-2^COVID^ add up to the PHQ-4^COVID^. In the present sample, for the medical students from UoN and from Charité the Cronbach’s alpha of the PHQ-4^COVID^ was 0.827 and 0.836.

### Medical students’ attitudes towards online learning during the COVID-19 pandemic

We measured the students’ attitudes towards online learning (OL) with 8 items by using a 5-point Likert scale ranging from 1 (strongly disagree) to 5 (strongly agree), it grants the option “don’t know”. For the items 1, 2, 3, 5, and 6 scoring with 1 indicates a negative evaluation and for the items 4, 7, and 8 it indicates a positive evaluation [5]. For this reason, we recorded the items 4, 7, and 8 in order to steer the meaning of the answers in the same direction as for the other items mentioned above. This step is needed to generate an OL Score (scale mean). The OL score ranges from 1 to 5, while lower values indicating students’ negative attitudes and higher values indicating students’ positive attitudes towards OL. In the present sample, for the medical students from UoN and from Charité the Cronbach’s alpha of the OL scale was 0.623 and 0.698. These are “questionable” to “acceptable” levels of internal consistency [12].

### Occupational stress and mental health during the COVID-19 pandemic

We used two open-end questions to detect coping mechanisms of the students and stress relieving activities There questions were *“Is there anything that you think has been helping your mental health and wellbeing during the COVID-19 pandemic?”* and *“Is there anything you have done to try to relieve stress during the COVID-19 pandemic?”* from a qualitative study by Tawse and Demou [13].

### Data analysis

Characteristics of students were presented as number and as percentage for categorical variables and as mean and standard deviation (SD) for the continuous variables. A χ²-test was used to compare socio-demographic characteristics and study location. Furthermore, a χ²-test for association was conducted between study location and beneficial factors for mental health and stress relieving activities during the COVID-19 pandemic. All expected cell frequencies were greater than 5. Reliability was calculated using Cronbach’s alpha. These coefficients were considered acceptable (α ≥ 0.70), good (α ≥ 0.80), or very high (α ≥ 0.90). Concurrent validity was evaluated with Spearman correlations between the OL Score and the PHQ-4^COVID^. Correlation coefficients were interpreted as absent (< 0.20), very weak (0.20 to < 0.40), weak (0.40 to < 0.60), moderate (0.60 to < 0.80), or strong (0.80 to < 1.0) [14]. We performed a content analysis of the structured open-end questions according to Kuckartz [15]. Furthermore, we used multiple regression analysis (with backwards method) to assess associations between the attitudes’ towards OL and PHQ-4^COVID^. Gender and year of study were used as sociodemographic covariates. Analyses were performed using IBM SPSS Statistics for Windows, version 27.0 (IBM Corp., Armonk, NY, USA). *P* values < 0.05 were considered to indicate statistical significance.

## Results

The sociodemographic data is shown in Table 1. Respondents were from Charité in Berlin (34.4%) or from the University of Nairobi (UoN) (65.5%). Both subsamples had more female (53.8% from Charité, 66.7% from UoN) than male participants (45.0%, 26.2% respectively). A few of the respondents (4.2%) from Germany identified as genderfluid. Results did not show a significant difference between gender and study location, χ²(3) = 7.34, *P* = 0.062. Majority of the respondents in Kenya (47.5%) were in year 5, while a majority of the students in Germany (54.8%) were in year 4. Results show a significant difference between year of study and study location, χ²(2) = 10.84, *P* = 0.004. The results show a difference in the living conditions between students at Charité and those from the UoN; 26.2% of students from Charité lived alone while only 5.0% of students from UoN, lived alone during the pandemic. Most students from UoN (93.8%) lived with their families during the pandemic while only 28.6% of students from Charité stayed with their families. Results show a significant difference between living condition and study location, χ²(3) = 59.05, *P* = 0.000. During the pandemic, the majority (78.8%) of the students in Kenya did not have part time jobs, with only 21.2% of the students having part time jobs (13.8% of the students had part time non-medical jobs, 6.3% of the students had part time jobs in healthcare institutions not related to COVID-19, and only 1.3% had a part time job in a healthcare institution related to COVID-19). In Germany, most (78.6%) of the students had part time jobs of which 38.1% had jobs in healthcare institutions related to COVID-19, 31% had part time jobs in healthcare institutions not related to COVID-19 while 9.5% had non-medical jobs during the pandemic. Only 21.4% of the students in Germany had no part time jobs during the pandemic. Results show a significant difference between having a part-time job and study location, χ²(3) = 53.96, *P* = 0.000. We further observed the differences in the two sub-populations in the differences in COVID-19 diagnosis. Students in Germany had more clinically confirmed cases of COVID-19 (71.4%) while in Kenyan only 10% of students were clinically confirmed with COVID-19. Only 9.5% of the German students were suspected to be infected by the COVID-19 virus, while 28.8% of the Kenyan students were asymptomatic or suspected. 61.3% of the students in Kenya had no diagnosis made of COVID-19 infection while 19% of the German students had no diagnosis made. Results show a significant difference between COVID-19 diagnosis and study location, χ²(3) = 48.46, *P* = 0.000.

The was no significant difference between levels of depression and anxiety as measured with PHQ-2^COVID^ and GAD-2^COVID^ sum scores between UoN and Charité students (*P* > 0.05) (Table 2). In the analysis for individual PHQ-2^COVID^ items, 23.8% of Kenyan students had scores of ≥ 3, while 21.43% of German students had scores of ≥ 3 and were thus predisposed to experiencing depression during the pandemic. The individual GAD-2^COVID^ items revealed that 27.5% of UoN students and 28.6% of the Charité students scored ≥ 3 and were thus predisposed to experiencing anxiety during the pandemic. Overall, the PHQ-4^COVID^ score did not differ between students from UoN and Charité (M = 4.21; SD = 2.69 and M = 3.93; SD = 2.63; *P* > 0.05, respectively). The results for the satisfaction with online learning (OL) are shown in Table 2. Most German students were less satisfied with the transition to OL during the COVID-19 pandemic (M = 2.97; SD = 1.36) compared to UoN students (M = 3.35; SD = 1.3; *P* < 0.05). UoN students were more satisfied by the effort their institution made to help them adapt to online learning OL 1 (M = 3.26, SD = 1.34) than the German students (M = 2.69; SD = 1.02; *P* < 0.05). Kenyan students reported a greater preference for online learning; OL 2 (M = 3.13; SD = 1.85) compared to the German students (M = 2.71; SD = 1.11; *P* < 0.05), indicating higher overall satisfaction. Most Kenyan students agreed that the acquisition of necessary gadgets to enable them take part in online learning (OL 4) became an added burden to them and their families (M = 3.05, SD = 1.02, *P* < 0.05). This was not the case for most German students. Similarly, in comparison to most German students (M = 2.71, SD = 1.11), most of the Kenyan students spent less money on additional food, housing and travel during the online learning phase (OL 6) due to the pandemic (M = 3.84, SD = 1.05; *P* < 0.05). Students from both countries observed that they started procrastinating more with the introduction of online learning modules (OL 8, *P* < 0.05). Overall, the attitude towards OL (OL Score) did not differ between students from both universities (M = 3.21; SD = 0.60 and M = 2.91; SD = 0.61; *P* > 0.05, respectively).

**Table 2:** Emotional distress and attitudes towards OL among the medical students from UoN and Charité during the COVID-19 pandemic (*N* = 122)

	UoN (*n* = 80) M (SD)	Charité (*n* = 42) M (SD)	*P*
PHQ-2 ^*COVID*^	2.12 (1.36)	2.02 (1.22)	0.960
GAD-2 ^*COVID*^	2.09 (1.54)	1.91 (1.59)	0.540
PHQ-4 ^COVID^	4.21 (2.69)	3.93 (2.63)	0.578
OL 1: I believe that our university is doing everything it can to help students adapt to OL during a pandemic	3.26 (1.34)	2.69 (1.02)	0.004
OL 2: Online learning is a more comfortable way of learning for me.	3.13 (1.85)	2.71 (1.11)	0.043
OL 3: During the OL period, I began to study more on my own.	2.90 (1.29)	3.02 (1.60)	0.297
OL 4: Buying the necessary gadgets to participate in OL has become an additional burden for me and my family.	3.05 (1.20)	2.10 (1.03)	0.001
OL 5: The absence of the need to change campuses and the training bases made my life easier.	3.50 (1.25)	3.21 (2.14)	0.323
OL 6: One of the positive features of OL is the possibility to spend less money on additional food, travel, and housing.	3.84 (1.05)	2.71 (1.11)	0.001
OL 7: With the transition to OL, I began to feel lonely.	3.29 (1.14)	3.74 (1.15)	0.161
OL 8: With the transition to OL, I began procrastinating more often.	3.84 (1.14)	3.55 (1.31)	0.032
OL Score*: scale mean	3.12 (0.60)	2.91 (0.61)	0.078

*PHQ-2 (range 0–6)*,* GAD-2 (range 0–6)*,* OL Score (range 1–5)*, *=items 4,*7*,*8 recoded*

**Fig. 1:**
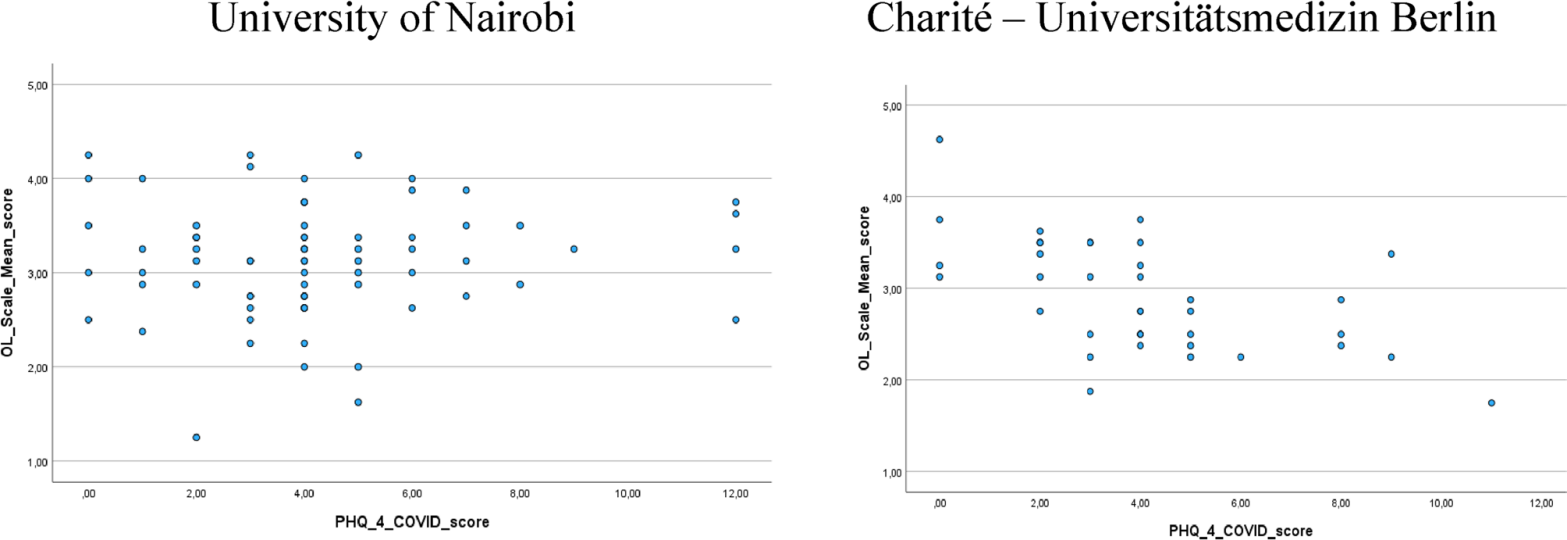
Scatter plot showing pairs of values for OL Score and PHQ-4^COVID^ for medical students at UoN (*n* = 74) and Charité (*n* = 38)

We identified no correlation between attitude towards OL (OL Score) during the COVID-19 pandemic and mental health symptoms regarding anxiety and depression (PHQ-4^COVID^) for students’ from UoN, r_s_ = 0.062, *P* > 0.05). However, in this regard there was a strong negative correlation for students’ from Charité, r_s_ = − 0.570, *P* < 0.001). Our findings indicate that the more positive the students’ attitudes towards OL was, the less burden regarding anxiety and depression German medical students reported (Fig. 1).

University of Nairobi Charité – Universitätsmedizin Berlin.

Regarding mental health during the pandemic, most students from both countries attributed social support from family, friends, and religious institutions as a benefactor in coping (UoN: 50.0%/Charité: 59.5%). This was followed by activities such as sporting and hobbies (36.3%) and social media (12.5%) for students from UoN. Only one student from UoN reported other activities such as occupation and living conditions (Table 3). Charité students (26.2%) attributed other factors such as Occupation and living conditions as a key beneficial factor to their coping mechanisms after Social support. A chi-square test was used to compare study location and beneficial factors for mental health during the COVID-19 pandemic. Results show a significant difference between both variables, χ²(3) = 27.52, *P* = 0.000, Cramer V = 0.475. Regarding stress relieving activities during the COVID-19 pandemic, most of the students from UoN (40.0%) identified with hobbies such as movies, reading, writing, and social media followed by sporting activities (36.3%) and lastly social support from their families, friends and religious institutions (21.3%). Most of the Charité students (33.3%) identified with sporting activities such as gym, walks and workouts followed by hobbies (28.6%) and lastly meditation and yoga (16.7%). (Table 3). A chi-square test was used to compare study location and stress relieving activities during the COVID-19 pandemic. Results show a significant difference between both variables, χ²(3) = 13.13, *p* = 0.022, Cramer V = 0.328.

**Table 3:** Beneficial factors for mental health and stress relieving activities assessed by open-end questions (*N* = 122)

Beneficial factors	UoN (*n* = 80) (*n*, %)	Charité (*n* = 42) (*n*, %)		*P*
Social support (family, friends, and religious institutions)	40 (50.0)	25 (59.5)		0.000
Sporting activities and hobbies	29 (36.3)	3 (7.1)		
Social media	10 (12.5)	3 (7.1)		
Others (occupation and living condition)	1 (1.3)	11 (26.2)		
Stress Relieving activities				
Hobbies (movies, reading, writing, social media)	32 (40.0)		12 (28.6)	0.022
Sporting activities (walks, gym, workout)	21 (26.3)		14 (33.3)	
Social support (family, friends & religious institutions)	17 (21.3)		2 (4.8)	
Meditation and yoga	6 (7.5)		7 (16.7)	
Coping mechanisms (eating, smoking, drinking, positive focus)	2 (2.5)		2 (4.8)	
Others (new languages, new activities etc.)	2 (2.5)		5 (11.9)	

For the UoN students, OL 3 (during the OL period, I began to study more on my own), OL 8 (with the transition to OL, I began procrastinating more often), and year of study were able to statistically significantly predict PHQ-4^COVID^, (*F* (3,70) = 3.859, *P* < 0.05) and for the Charité students this applies to OL 3 (during the OL period, I began to study more on my own) and OL 7 (with the transition to OL, I began to feel lonely) (*F*(2,35) = 8.940, *P* < 0.001). In Table 4, further findings for UoN show, if agreement to OL 3 increases by one point, the PHQ-4^COVID^ increases by 0.611 units. These findings suggest that during the OL period, increased independent UoN medical was associated with a higher psychological distress. Moreover, if students began procrastinating more often, they also experienced more mental health symptoms (*b* = 0.693, *P* = 0.012) and with every year of study, symptom burden also increased (*b* = 0.925, *P* = 0.041). For UoN students, the R^2^ for the overall model was 0.142 (adjusted R^2^ = 0.105), indicative of a moderate goodness-of-fit according to Cohen [16]. Findings also show that 10.5% of the total dispersion of the PHQ-4^COVID^ is explained by these three variables. Findings for Charité students’ show, for each one-point increase in agreement with OL 3, PHQ-4^COVID^ scores decreased by −1.134 units. This finding indicated that during the OL period, greater independent study was associated with a lower symptom burden among this group (*P* < 0.001). Among German medical students, higher levels of procrastination were significantly associated with increased psychological distress (*b* = 0.723, *P* = 0.046). Regression analysis did not identify year of study as relevant for the overall model among students at Charité. For Charité students, the R^2^ for the overall model was 0.338 (adjusted R^2^ = 0.300), indicative for a high goodness-of-fit according to Cohen [16]. Findings also show that 30.0% of the total dispersion of the PHQ-4^COVID^ is explained by these two variables.

**Table 4:** Parameter estimator of the regression model for the PHQ-4^COVID^

Independent variables	b	SE	t	Sig.
***UoN***				
Constant	−2.056	1.899	−1.083	0.283
OL 3: During the OL period, I began to study more on my own.	0.611	0.257	2.380	0.020
OL 8: With the transition to OL, I began procrastinating more often.	0.693	0.270	2.569	0.012
Year of study	0.925	0.444	2.084	0.041
Charité				
Constant	4.494	1.742	2.842	0.007
OL 3: During the OL period, I began to study more on my own.	−1.134	0.308	−3.681	< 0.001
OL 7: With the transition to OL, I began to feel lonely.	0.723	0.349	2.069	0.046

*b = regression coefficient*,* SE = standard error*

## Discussion

Student satisfaction with the introduction of online learning during the COVID-19 pandemic was influenced by multiple factors, including individual coping strategies. When faced with pandemic-related challenges, students employed different coping mechanisms, which likely impacted their mental health outcomes [17]. The depression and anxiety standard scores used in this study, namely the PHQ-2^COVID^ and GAD-2^COVID^ sum scores, revealed higher levels of depression among Kenyan students compared to their German counterparts. Given the ‘acceptable’ to ‘good’ levels of internal consistency [12], these ultra-brief screening instruments provide a reliable measure of depression and anxiety symptoms among students. Furthermore, the adoption of validated scales, such as PHQ-2^COVID^ and GAD-2^COVID^, is a widely accepted practice in survey research [18], ensuring consistency and comparability of findings across different populations. The pandemic was associated with an economic depression in most economies, loss of jobs, travel restrictions and isolation [19]. These could have largely contributed to anxiety and depression among adults and students alike [20]. Data from our study show that most German medical students were less satisfied with the transition to Online Learning compared to their Kenyan counterparts. The latter acknowledged institutional efforts to support their adaptation to the new learning model. Additionally, a greater proportion of Kenyan students expressed a preference for online platforms for their learning experience relative to the German students. The preference by Kenyan students could be attributed to the excitement brought about by new learning tools as well as the convenience it offered as shown in a case study by Gustiani [21]. The variability with the Germans could be attributed to multiple online distractions associated with online platforms, lack of face-face interactions with their instructors as well as the possibility of receiving non-bedside clinical training and experience from patients and laboratories [22]. Compared to German medical students, most Kenyan students found it more burdensome to acquire devices necessary for online learning. This could possibly be due to the low socioeconomic status of most Kenyan students as seen in a systematic review of health science students experience with online learning during the pandemic [23]. They however spent less money on additional food, housing and travel as they relocated back to their parents mostly in rural Kenya [24]. Studies in other parts of the world such as Jordan and India showed that lack of electricity and poor infrastructure in remote areas limited the access to online learning by students from such regions [25]. Equally, some families had no financial ability to purchase laptops, cell phones and incur the cost of installing internet connectivity to enable the online learning process of their children [23]. Students from both UoN and Charité pointed to procrastination as a downside of the online learning model which significantly increased the symptom burden of depression and anxiety [20]. Loneliness was associated with greater psychological distress related to depression and anxiety among students in UoN and Charité. Similarly, students who started studying on their own without the presence of their classmates from both UoN and Charité had higher risks of developing symptoms of anxiety and depression [26]. Coping with the challenges of the pandemic was largely attributed to social support from family, friends, and religious organizations. As social beings, the students’ interactions with different people gave them a platform to share their grievances and seek help which proved vital for their mental health [27]. They equally found sporting and hobbies as a coping technique followed by social media. According to Cauberghe et al. [2], young adults spent most of their time during the pandemic on social media platforms for humor and interaction with their friends as a coping technique during the period [2]. In Kenya, most students resorted to hobbies such as movies, writing, reading and social media as stress relieving mechanisms while a majority of German students identified with sporting activities that includes gym, workouts and walks. Fullana et al. [28], in their study during the pandemic attributed enjoyable leisure activities to improve the psychosocial and mental health of individuals during the pandemic. As such, these leisure activities and hobbies significantly improved the well-being of individuals and reduced the anxiety and depressive symptoms significantly [28]. A few of German students equally resorted to Meditation and Yoga as a coping strategy.

### Limitations and suggestions for future research

To our knowledge, this was the first pilot study among a sample of medical students from two institutions, UoN and Charité, considering experiences and mental wellbeing regarding OL during the COVID-19 pandemic. The web-based survey enabled us to collect standardized responses from 80 medical students at UoN and 42 students at the Charité, all in their 4th to 6th year of study in 2023 at the time of data collection. However, the study’s limitations need to be addressed. The present study was designed as a comparative cross-sectional study, as a result of which no causalities can be determined. It is a convenience sample. Thus, it limits external validity and the generalizability of findings for medical students in Kenya and in Germany. A representative sample would require a central study register that is accessible for research purposes in order to gain access to the entire population of medical students in both countries to be able to draw a representative sample. Moreover, another limitation of this study is the self-reported nature of the responses which rely on the students’ recall on a past period specified as during the COVID-19 pandemic. Furthermore, while the structure and content of medical curricula in both countries differ, a detailed curriculum comparison was beyond the scope of this study. As a limitation, our assessments of depression and anxiety can only be interpreted as initial indications of students’ situation during a pandemic. Clinically relevant screening was beyond the scope of this study. Future research should focus on the need to have a restructuring of the curricular in case of online transitions, pre-pandemic installation of mentorship and digital formats to evaluate the mental health and monitor academic wellbeing of learners. Moreover, we recommend a long term follow up in future systematic longitudinal studies to evaluate the long term academic and clinical impact of COVID-19 to learners. 

## Conclusion

The cross-cultural survey between Kenya and Germany reports a significant impact on both mental health and medical training among undergraduate medical students during the COVID-19 pandemic. The online learning model that substituted face to face learning during the COVID-19 pandemic, a global public health emergency, had both benefits and setbacks alike in both settings. The lives of students were affected by changes in the curriculum to suit the new learning model such as pre-recorded lectures, telemedicine and virtual clinical case discussions as opposed to bedside case presentations. The changes equally affected the interpersonal relationship between learners and their tutors that is exemplified in face to face interactions, there were financial implications with acquisition of tools to support the new model and some students contracted the virus. Students from both countries experienced marked anxiety and depression. These findings could be useful in enhancing changes that would ensure standardized medical education at a time of widespread unprecedented pandemics like COVID-19 in future. Digital tools could also be put in place for the provision of both academic and psychological support to learners at such times.

## Data Availability

Data will be publicly shared upon acceptance. Data can be requested through contacting the corresponding author.

## Abbreviations

MBChB: Bachelor of medicine
UoN: University of Nairobi
OL: Online Learning
WHO: World health organization
SD: Standard deviation

## References

[1] Chakladar J, Diomino A, Li WT, et al. Medical student’s perception of the COVID-19 pandemic effect on their education and well-being: a cross-sectional survey in the united States. BMC Med Educ. 2022;22:1–10. 10.1186/s12909-022-03197-x. 35248030 10.1186/s12909-022-03197-x PMC8897763

[2] Struthers DR, Allsop Y, Kalelioğlu F, Rzyankina E. The impact of COVID-19 on higher education: A systematic literature review of pedagogical approaches and challenges. In: Auer ME, Pester A, May D, editors. Learning with technologies and technologies in learning: Experience, trends and challenges in higher education. Springer; 2022. pp. 367–90.

[3] Alsoufi A, Alsuyihili A, Msherghi A, Elhadi A, Atiyah H, Ashini A, et al. Impact of the COVID-19 pandemic on medical education: medical students’ knowledge, attitudes, and practices regarding electronic learning. PLoS ONE. 2020;15:e0242905. 10.1371/journal.pone.0242905. 33237962 10.1371/journal.pone.0242905 PMC7688124

[4] Rajab MH, Gazal AM, Alkattan K. Challenges to online medical education during the COVID-19 pandemic. Cureus. 2020;12:e8966. 10.7759/cureus.8966. 32766008 10.7759/cureus.8966 PMC7398724

[5] Bolatov AK, Seisembekov TZ, Dauyenov E, Zhorokpayeva MD, Smailova DS, Pavalkis D. Medical education during the coronavirus disease pandemic and students’ mental health: a one-year follow- up. Front Ed. 2022;7:1–14. 10.3389/feduc.2022.1025600.

[6] Guse J, Heinen I, Mohr S, Bergelt C. Understanding mental burden and factors associated with study worries among undergraduate medical students during the Covid-19 pandemic. Front Psychol. 2021;12:734264. 10.3389/fpsyg.2021.734264. 34975624 10.3389/fpsyg.2021.734264 PMC8714644

[7] Wafula I, Ong’era EM. Deprived of the sea: being a Kenyan Final-year medical student during the COVID-19 outbreak. IJMS. 2021;9:80–1. 10.5195/ijms.2021.692.

[8] Bellows S, Rosalie HALL. Ethnicity and culture in Kenyan cooperatives. Afr J Co-Op Dev Technol. 2018;3:11–29. 10.58547/1.v3i1.15.

[9] Darwish AF, Huber G. Individualism vs collectivism in different cultures: a cross-cultural study. Intercult Educ. 2003;14:47–56. 10.1080/1467598032000044647.

[10] Kroenke K, Spitzer RL, Williams JB. The patient health questionnaire-2: validity of a two-item depression screener. Med Care. 2003;41:1284–92. 10.1097/01.MLR.0000093487.78664.3C. 14583691 10.1097/01.MLR.0000093487.78664.3C

[11] Kroenke K, Spitzer RL, Williams JBW, Löwe B. An ultra-brief screening scale for anxiety and depression: the PHQ-4. Psychosomatics. 2009;50:613–21. 10.1176/appi.psy.50.6.613. 19996233 10.1176/appi.psy.50.6.613

[12] George D, Mallery P. SPSS for windows step by step. A simple guide and reference. 12rd ed. Boston: Allyn and Bacon; 2005.

[13] Tawse J, Demou E. Qualitative study to explore UK medical students’ and junior doctors’ experiences of occupational stress and mental health during the COVID-19 pandemic. BMJ Open. 2022;12:e065639. 10.1136/bmjopen-2022-065639. 10.1136/bmjopen-2022-065639 PMC9748513 36523252

[14] Field A. Discovering statistics using IBM SPSS statistics. London: SAGE; 2018.

[15] Kuckartz U. Qualitative text analysis: A systematic approach. In: Kaiser G, Presmeg N, editors. Compendium for early career researchers in mathematics education. Heidelberg: Springer; 2019. pp. 181–97. 10.1007/978-3-030-15636-7_8.

[16] Cohen J. Statistical power analysis for the behavioral sciences. Hillsdale: L. Erlbaum Associates; 1988.

[17] Pokhrel S, Chhetri R. A literature review on impact of COVID-19 pandemic on teaching and learning. High Educ Future. 2021;8:133–41. 10.1177/2347631120983481.

[18] Furr RM. Scale construction and psychometrics for social and personality psychology. 2011. 10.4135/9781446287866

[19] Godinić D, Obrenovic B. Effects of economic uncertainty on mental health in the COVID-19 pandemic context: social identity disturbance, job uncertainty and psychological well-being model. IJED. 2020;6:61–74. 10.18775/ijied.1849-7551-7020.2015.61.2005.

[20] Fawaz M, Samaha A. E-learning: depression, anxiety, and stress symptomatology among Lebanese university students during COVID‐19 quarantine. Nurs Forum. 2021;56:52–7. 10.1111/nuf.12521. 33125744 10.1111/nuf.12521

[21] Gustiani S. Students’ motivation in online learning during COVID-19 pandemic era: a case study. Holsitics. 2020;12:23–40.

[22] Heitmann H, Wagner P, Fischer E, Gartmeier M, Schmidt-Graf F. Effectiveness of non-bedside teaching during the COVID-19 pandemic: a quasi-experimental study. BMC Med Educ. 2022;22:1–7. 10.1186/s12909-022-03141-z. 35101016 10.1186/s12909-022-03141-z PMC8801559

[23] Abdull Mutalib AA, Md. Akim A, Jaafar MH. A systematic review of health sciences students’ online learning during the COVID-19 pandemic. BMC Med Educ. 2022;22:524. 10.1186/s12909-022-03579-1. 35786374 10.1186/s12909-022-03579-1 PMC9251028

[24] Sindiani AM, Obeidat N, Alshdaifat E, Elsalem L, Alwani MM, Rawashdeh H, et al. Distance education during the COVID-19 outbreak: a cross-sectional study among medical students in North of Jordan. Ann Med Surg. 2020;59:186–94. 10.1016/j.amsu.2020.09.036. 10.1016/j.amsu.2020.09.036 PMC7531436 33042535

[25] Aljaraideh Y, Al Bataineh K. Jordanian students’ barriers of utilizing online learning: a survey study. Int Educ Stud. 2019;12:99–108. 10.5539/ies.v12n5p99.

[26] Wong SYS, Zhang D, Sit RWS, Yip BHK, Chung RY, Wong CKM, Chan DCC, Sun W, Kwok KO, Mercer SW. Impact of COVID-19 on loneliness, mental health, and health service utilisation: a prospective cohort study of older adults with Multimorbidity in primary care. Br J Gen Pract. 2020;70:e817–24. 10.3399/bjgp20X713021. 32988955 10.3399/bjgp20X713021 PMC7523921

[27] Aristovnik A, Keržič D, Ravšelj D, Tomaževič N, Umek L. Impacts of the COVID-19 pandemic on life of higher education students: a global perspective. Sustainability. 2020;12:1–34. 10.3390/su12208438.35136666

[28] Fullana MA, Hidalgo-Mazzei D, Vieta E, Radua J. Coping behaviors associated with decreased anxiety and depressive symptoms during the COVID-19 pandemic and lockdown. J Affect Disord. 2020;275:80–1. 10.1016/j.jad.2020.06.027. 32658829 10.1016/j.jad.2020.06.027 PMC7329680

